# A Mother’s struggle: postpartum preeclampsia complicated by triple vessel spontaneous coronary artery dissection

**DOI:** 10.1093/omcr/omae120

**Published:** 2024-10-15

**Authors:** Branco G M Bettinotti, Mickias B Tegegn, Victor Razuk, Som Bailey, Rowena Hann, Cesar Mendoza, Marian T Calfa, Rosario A Colombo

**Affiliations:** Department of Internal Medicine, Mount Sinai Medical Center, 4300 Alton Rd, Miami, FL 33140, United States; Department of Medicine, University of Miami Miller School of Medicine, 1600 NW 10th Ave, Miami, FL 33136, United States; Cardiovascular Division, Department of Medicine, 1600 NW 10th Ave, University of Miami Miller School of Medicine, Miami, FL 33136, United States; Interventional Cardiology, 1600 NW 10th Ave, University of Miami Miller School of Medicine, Miami, FL 33136, United States; Department of Medicine, University of Miami Miller School of Medicine, 1600 NW 10th Ave, Miami, FL 33136, United States; Interventional Cardiology, 1611 NW 12th Ave, Jackson Memorial Hospital, Miami, FL 33136, United States; Department of Medicine, Cardiovascular Diseases Division, 1611 NW 12th Ave, Jackson Memorial Hospital, Miami, FL 33136, United States; Cardiovascular Division, Department of Medicine, 1600 NW 10th Ave, University of Miami Miller School of Medicine, Miami, FL 33136, United States; Cardiovascular Division, Department of Medicine, Jackson Memorial Hospital, 1611 NW 12th Ave, Miami, FL 33136, United States

**Keywords:** cardiology and cardiovascular systems, medical disorders in pregnancy, emergency medicine

## Abstract

Spontaneous coronary artery dissection (SCAD) is an important cause of acute coronary syndrome among young women, especially in the postpartum period. Pregnancy-Associated SCAD (P-SCAD), an aggressive subtype, frequently involves multi-vessel dissection, decreased left ventricular function, and higher mortality. Here we present a rare case of postpartum pre-eclampsia complicated by multi-vessel SCAD in a 40-year-old multiparous Haitian black woman. Diagnosis was established with coronary angiography which revealed spontaneous dissection of the left anterior descendant, left circumflex, and right coronary arteries. Given the patient remained hemodynamically stable no percutaneous coronary intervention was indicated. She experienced recurrent anginal symptoms during her hospitalization that were managed with the addition of clopidogrel. The pathophysiology of P-SCAD is not well understood and thought to be related to an increased state of hemodynamic stress and hormonal fluctuation. The role of pre-eclampsia as a risk factor remains poorly defined and warrants further investigation.

## Introduction

Spontaneous coronary artery dissection (SCAD) is a rare cause of acute coronary syndrome (ACS), affecting an estimated 4% of patients presenting with ACS [1].

Well-established risk factors include female gender, fibromuscular dysplasia (FMD), and pregnancy [2].

Pregnancy-associated SCAD (P-SCAD) describes cases affecting women during, or shortly after pregnancy. This accounts for over 40% of pregnancy-associated myocardial infarctions and up to 17% of all SCAD cases [1]. P-SCAD is further associated with multiparity, preeclampsia, and exposure to hormonal therapy [3].

Delayed postpartum preeclampsia is defined as that with onset from 2 days to 6 weeks following delivery [4].

We present the first case, to our knowledge, of a 40-year-old female with delayed postpartum preeclampsia complicated by an ST elevation myocardial infarction (STEMI) due to triple-vessel SCAD.

## Case report

**Figure 1:**
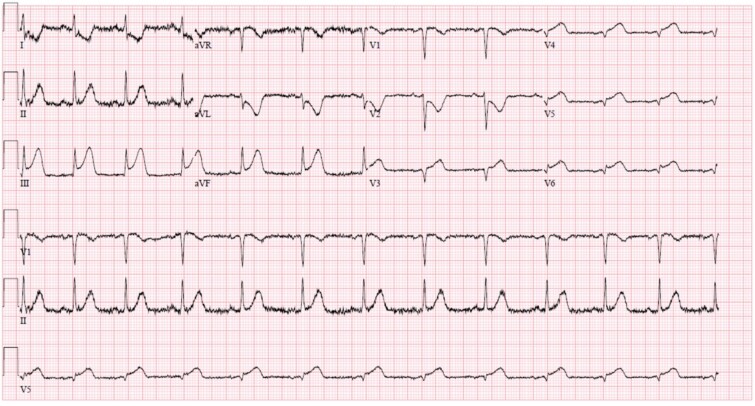
Admission electrocardiogram obtained during the first chest pain episode. Note the ST segment elevation on leads III, aVF, V4, V5 & V6 and reciprocal changes.

**Figure 2:**
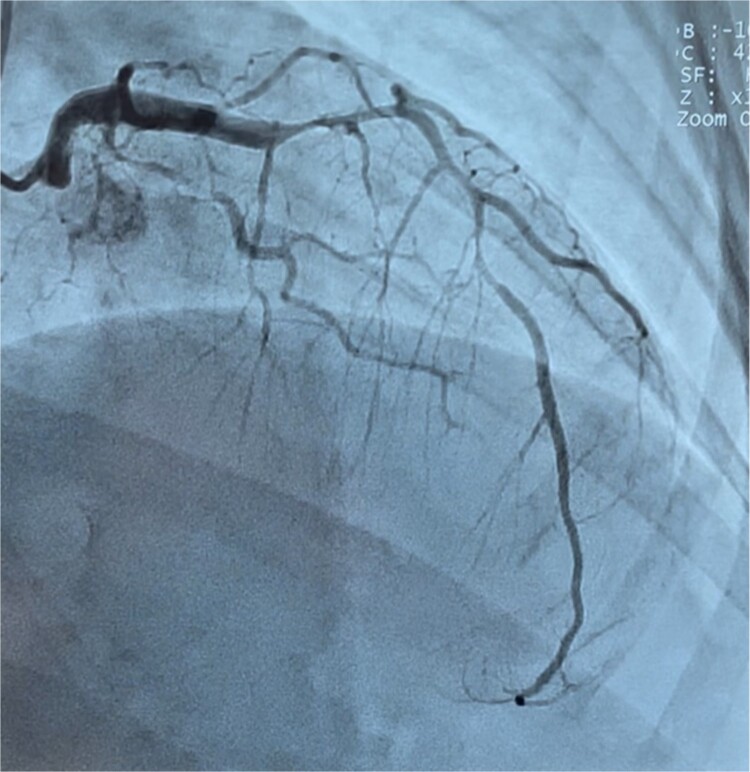
Coronary angiography demonstrating multiple focal areas of dissection involving the proximal left anterior descending artery and proximal left circumflex with extension distally in both arteries.

**Figure 3:**
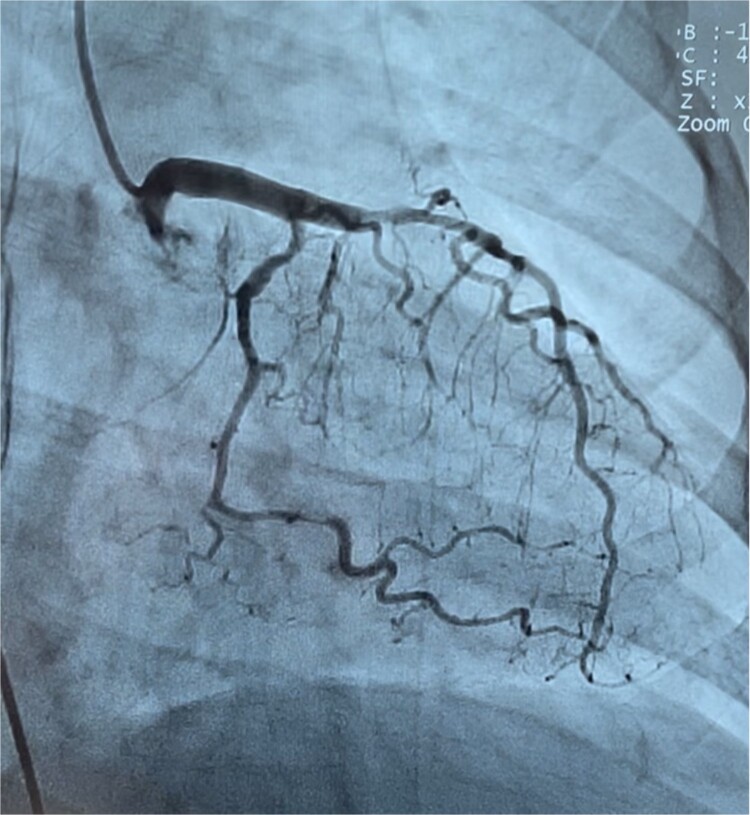
Coronary angiography demonstrating focal areas of dissection at the ostium and distal portion of the mid left circumflex coronary artery.

**Figure 4:**
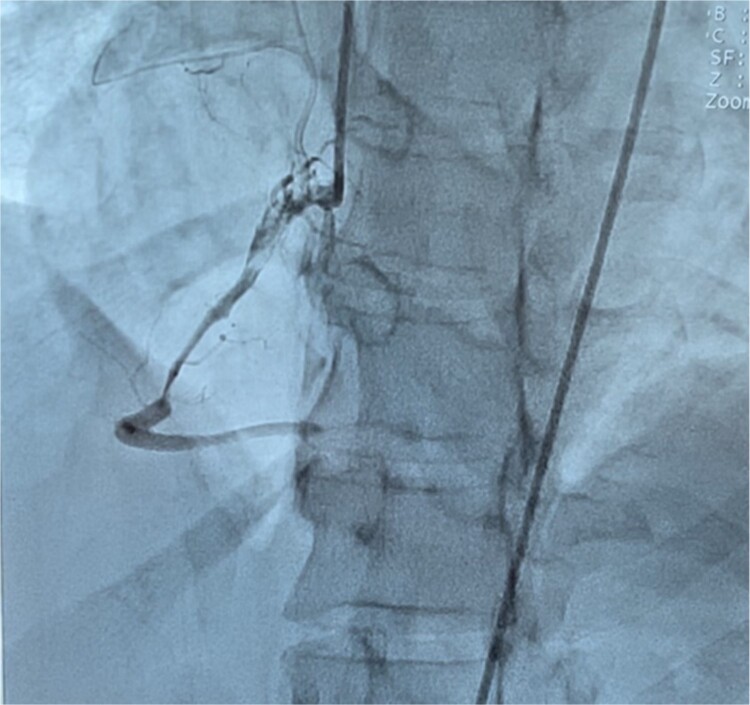
Coronary angiography demonstrating a long spiral dissection originating in the ostium and extending to the mid-portion of the right coronary artery.

A 40-year-old multiparous Haitian black woman with no known cardiovascular risk factors, and a history of two prior miscarriages underwent a full-term vaginal delivery, and was discharged after an uneventful postpartum course. On postpartum day six, she was briefly re-admitted for postpartum preeclampsia. She denied any personal history of drug, tobacco or alcohol use, or hormonal treatment. On postpartum day eleven, she returned to the emergency department complaining of sudden onset, left-sided, sub-sternal chest tightness radiating to her left arm, shortness of breath, and diaphoresis. A physical exam in the emergency room revealed blood pressure of 122/78 mmHg, heart rate 112 beats per minute, and oxygen saturation of 100% on 2-liters nasal cannula. There was no appreciable cardiac murmur, rales, jugular venous distention, or peripheral edema. Chest x-ray was unremarkable. 12-lead EKG (Fig. 1) showed 1 to 3 mm ST segment elevations in leads II, III, aVF, V4, V5, and V6 consistent with inferolateral STEMI. Troponin-I levels peaked at 78.5 ng/ml (N: 0–0.034 ng/ml). Lipid profile revealed total cholesterol of 269 mg/dl, LDL of 187 mg/dl, and HDL of 54 mg/dl. Transthoracic echocardiogram (TTE) showed left ventricular ejection fraction (LVEF) of 50%–55%, and moderate hypokinesis of inferolateral, inferior, and mid-anteroseptal walls. Coronary angiography revealed dissections of the left anterior descending (LAD) (Fig. 2) and left circumflex (LCx) arteries (Fig. 3), and a long spiral dissection of the right coronary artery (RCA) (Fig. 4). Intravascular ultrasound was not performed to avoid the risk of subintimal wire positioning and worsening of coronary artery dissection.

Given her hemodynamic stability, resolution of chest pain, and TIMI 3 flow in all coronary vessels; no percutaneous revascularization was pursued. We initiated conservative medical therapy with metoprolol succinate, aspirin, and nitroglycerin drip for angina relief. On the fourth day after admission, the patient developed epigastric pain with pericardial features, not relieved by nitroglycerin, without ECG changes and an initial down-trending troponin I of 1.16 ng/ml, suspicious for peri-infarction pericarditis; colchicine was started. Repeat TTE showed unchanged LVEF. A rebound in troponin I to 5.32 ng/ml prompted our decision to add clopidogrel. There were no signs of extra-coronary vascular abnormalities on CTA of the neck, chest, abdomen and pelvis, ruling out FMD and other large-vessel vasculitides. She remained stable and was discharged home on day 8 with plans to continue clopidogrel for 4 weeks, and aspirin for at least 1 year. Follow-up 6 months later demonstrated a LVEF of 55% on TTE and no recurrent symptoms.

## Discussion

We present, to our knowledge, the first reported case of triple vessel peripartum SCAD in a patient with delayed postpartum preeclampsia who presented with an inferolateral STEMI involving all major coronary artery branches (LAD, LCx, RCA).

The most accepted pathophysiologic mechanism of SCAD is the “outside-in” theory, where the formation of an intramural hematoma arises de novo in the media, expanding, and occluding the lumen to finally tear the intimal wall and decompress. A proposed pathophysiologic mechanism that would explain P-SCAD media weakening is that progesterone inhibits collagen synthesis, while estrogen increases matrix metalloproteinases [5]. Importantly, 3 out of 4 SCAD cases present as STEMI and have higher rates of adverse outcomes such as urgent revascularization, mechanical support requirements, cardiogenic shock, ventricular tachycardia or fibrillation, and maternal and fetal mortality [1, 5, 6].

Multi-vessel involvement is when simultaneous acute dissections affect more than one artery without continuity between the involved vessels [1].

A comparative retrospective analysis of a SCAD registry revealed that most (70%) P-SCAD cases presented in the first month postpartum, over half (54%) of them within the first week. Those in the P-SCAD group had higher rates of STEMI (57% vs 36%; *P* = 0.009) and over 2-fold higher multi-vessel involvement (33% vs 14%; *P* = 0.0027). Less than half (41%) of patients in the first group were managed conservatively. There were no differences in 5-year recurrence rates. Notably, the study identified increased rates of preeclampsia in patients with P-SCAD (11%) compared to the 3.4% estimated rate in the general U.S. population [3].

Saw et al. described that peripartum SCAD up to 12 months from delivery is an independent predictor of major adverse cardiovascular events to 3 years (HR: 2.17). Recurrent chest pain is the most common symptom after SCAD, affecting 50.4% of patients at 1-month post-discharge and almost one-in-three patients (31.8%) at 3-year follow-up [7].

In stable patients the preferred treatment is conservative management with long-term aspirin and beta blockers, clopidogrel may be added to patients with high-grade stenosis [8]. In a multivariate analysis, only long-term Beta-blocker use (HR:0.36; *P* = 0.004) was associated with a decreased risk of recurrent SCAD at 3 years. Hypertension (HR:2.28; *P* = 0.019), however, was linked with an increased risk of recurrence [9].

Expert consensus on indications for percutaneous coronary intervention (PCI) are significant left main coronary artery (LM) disease, persistent angina, cardiogenic shock, TIMI grade 0 or 1 flow, and/or when medical management has failed. Coronary artery bypass grafting should be reserved for patients who underwent a failed PCI [1]. To this date there are no published results of any randomized control trial comparing treatment options. Most conservatively managed asymptomatic patients will demonstrate resolution of SCAD on follow-up angiography [10].

P-SCAD is a well-established entity with significant morbidity. This case highlights the importance of considering SCAD as part of the differential diagnosis in any pregnant or peripartum women presenting with acute chest pain.

## References

[1] Hayes SN , Tweet MS, Adlam D. et al. Spontaneous coronary artery dissection. J Am Coll Cardiol 2020;76:961–84. 10.1016/j.jacc.2020.05.084. 32819471

[2] Saw J , Aymong E, Sedlak T. et al. Spontaneous coronary artery dissection: association with predisposing Arteriopathies and precipitating stressors and cardiovascular outcomes. Circ Cardiovasc Interv 2014;7:645–55. 10.1161/CIRCINTERVENTIONS.114.001760. 25294399

[3] Tweet MS , Hayes SN, Codsi E. et al. Spontaneous coronary artery dissection associated with pregnancy. J Am Coll Cardiol 2017;70:426–35. 10.1016/j.jacc.2017.05.055. 28728686

[4] Al-Safi Z , Imudia AN, Filetti LC. et al. Delayed postpartum preeclampsia and eclampsia: demographics, clinical course, and complications. Obstet Gynecol 2011;118:1102–7. 10.1097/AOG.0b013e318231934c. 21979459

[5] Seecheran R , Kawall J, Ramadhin D. et al. Preeclampsia-associated multi-vessel spontaneous coronary artery dissection. J Investig Med High Impact Case Rep 2019;7:232470961987462. 10.1177/2324709619874624. PMC6740047 31509019

[6] Havakuk O , Goland S, Mehra A. et al. Pregnancy and the risk of spontaneous coronary artery dissection: an analysis of 120 contemporary cases. Circ Cardiovasc Inte 2017;10:e004941. 10.1161/circinterventions.117.004941. 28302642

[7] Saw J , Starovoytov A, Aymong E. et al. Canadian spontaneous coronary artery dissection cohort study. J Am Coll Cardiol 2022;80:1585–97. 10.1016/j.jacc.2022.08.759. 36265953

[8] Adlam D , Alfonso F, Maas AH. et al. European Society of Cardiology, acute cardiovascular care association, SCAD study group: a position paper on spontaneous coronary artery dissection. Eur Heart J 2018;39:3353–68. 10.1093/eurheartj/ehy080. 29481627 PMC6148526

[9] Saw J , Humphries KH, Aymong E. et al. Spontaneous coronary artery dissection. J Am Coll Cardiol 2017;70:1148–58. 10.1016/j.jacc.2017.06.053. 28838364

[10] Rogowski S , Maeder MT, Weilenmann D. et al. Spontaneous coronary artery dissection. Catheter Cardiovasc Interv 2017;89:59–68. 10.1002/ccd.26383. 26708825

